# Water Dynamics on Germinating Diaspores: Physiological Perspectives from Biophysical Measurements

**DOI:** 10.34133/2020/5196176

**Published:** 2020-12-06

**Authors:** J. P. Ribeiro-Oliveira, M. A. Ranal, M. A. Boselli

**Affiliations:** ^1^Instituto de Ciências Agrárias, Universidade Federal de Uberlândia, Avenida João Naves de Ávila, 2121, 38400-902 Uberlândia, Minas Gerais, Brazil; ^2^Instituto de Biologia, Universidade Federal de Uberlândia, Avenida João Naves de Ávila, 2121, 38400-902 Uberlândia, Minas Gerais, Brazil; ^3^Instituto de Física, Universidade Federal de Uberlândia, Avenida João Naves de Ávila, 2121, 38400-902 Uberlândia, Minas Gerais, Brazil

## Abstract

We demonstrated that classical biophysical measurements of water dynamics on germinating diaspores (seeds and other dispersal units) can improve the understanding of the germination process in a simpler, safer, and newer way. This was done using diaspores of cultivated species as a biological model. To calculate the water dynamics measurements (weighted mass, initial diffusion coefficient, velocity, and acceleration), we used the mass of diaspores recorded over germination time. Weighted mass of germinating diaspores has a similar pattern, independent of the physiological quality, species, or genetic improvement degree. However, the initial diffusion coefficient (related to imbibition *per se*), velocity, and acceleration (related to the whole germination metabolism) are influenced by species characters, highlighting the degree of genetic improvement and physiological quality. Changes in the inflection of velocity curves demonstrated each phase of germination *sensu stricto*. There is no pattern related to the number of these phases, which could range between three and six. Regression models can demonstrate initial velocity and velocity increments for each phase, giving an idea of the management of germinative metabolism. Our finds demonstrated that germination is a polyphasic process with a species-specific pattern but still set by the degree of genetic improvement and (or) physiological quality of diaspores. Among the biophysical measurements, velocity has the greatest potential to define the germination metabolism.

## 1. Introduction

The water flux from soil to diaspore (seeds and other dispersal units) is the main regulating factor for the end of anabiosis and, hence, for embryo growth and development. Thus, this relation plays a central role in diaspore water relations studies [1]. For a long time, water relations in the germination process were algebraically evaluated by means of the Lockhart model [2, 3], which describes the growth of single cells [4]. However, since germination is generally considered a binomial phenomenon (it either occurs or not), it is unfeasible to use the Lockhart model without modifications [3]. From this, several models were proposed to explain the influx and distribution of water in germinating diaspores in relation to temperature, water potential, irradiance, and plant hormones [3, 5–8]. These models are very important to predict diaspore germination behavior in field conditions, mainly under stress, but their complexity hinders their wider use by most diaspore scientists. This has led us to the following question. How can we better explore water dynamics on germinating diaspores in an easier and safer way?

Studies focusing on the kinetics of water on germinating diaspores seem to be a good answer. This biophysical approach has been previously proposed [9–13]. However, this approach only considers three phases of physiological activity associated with three steps of the water dynamics on germinating diaspores ([14] sense). This theory led scientists to a static idea that water dynamics on germinating diaspores is a quite defined process with three stages (phases). In practice, these stages can be observed by a curve that demonstrates the cumulative mass associated with tissue hydration in diaspores, inferring how the germination process occurs over time (see [15–17]). Thus, in classical literature [15, 16, 18–20], these phases are used by researchers as chronological markers of germination *sensu stricto*. This technique is very important for studies focusing on physiological, molecular, and morphophysiological aspects (e.g., [3, 21–29]), but it is poorly discussed. For instance, we saw that even when using just diaspore mass variation, it is possible to propose other models than the classical, as in the study of germination-dormancy balance in *Sisymbrium officinale* (L.) Scop. seeds [30].

The well-founded relation of germination and water content is based on physical and metabolic changes triggered by the water influx and managed by the embryo for its development [15, 16, 18–20]. The water influx from the environment to the germinating diaspore determines the embryo development potential since it is associated with maintaining cellular turgor and, hence, cell wall properties [3, 31], essential to growth and differentiation. Furthermore, it is also well known that water influx is conducted by alterations in the proportion of anabolism and catabolism in diaspore cells, which provokes fluctuations in the osmotic potential [17, 24]. These fluctuations alter diaspore mass over time. Taking this into account, we hypothesized that by measuring water dynamics on germinating diaspores accurately by means of classical biophysical measurements calculated from individual mass fluctuations, we will improve the understanding of germination *sensu stricto*. In any case, although diaspore mass is a continuous character, its fluctuations are associated with water potential, metabolic activity, and, therefore, the physiological process *per se*.

It is worth noting that in addition to the cell structure being progressively modified by water distribution in germinating diaspores [32, 33], the hormonal metabolism and oxidative metabolism are also triggered [34, 35]. This is the basis for deeper physiological changes promoted on germinating diaspores. Thus, an exact analysis of water intake and its movement during germination is necessary to understand all processes of life activation from anabiosis [33, 35, 36] to embryo protrusion. Nevertheless, the techniques used to perform this analysis, such as nuclear magnetic resonance [35, 36] and micromagnetic resonance imaging [32], are very expensive and require great technical support. On the other hand, mass evaluation can be a simple alternative to observe water distribution in germinating diaspores and has been classically used by diaspore scientists to support their hypotheses about water relations (e.g., [10, 12]). In this context, there are no doubts that inferences about the germination process from water dynamics on germinating diaspores are possible with little infrastructure. This would be useful for diaspore scientists acquiring information on the germinative metabolism from the start. As a consequence, they would not need to evaluate the process through destructive tests, such as the tetrazolium test, or infer germination only when it ends (embryo protrusion). In this consensus, we demonstrate how water dynamics on germinating diaspores can be used as a physiological and metabolic trait when studied by means of biophysical measurements associated with the increment of weighted mass.

Because of the aforementioned, we are proposing a new analytical approach for diaspore scientists to perform robust inferences on diaspore germination even with little infrastructure, without replacing classical and (or) contemporaneous measurements used to infer germination and (or) metabolism. When you perform a scenario transposition, it is possible to remember that in the early days, germination studies were based on cumulative curves [37], which allowed little accuracy and assessed the germination process indirectly. For this reason, several researchers have resorted to mathematical expressions to calculate uniformity, time, and velocity of germination accurately based on embryo protrusion [37]. Based on this assumption, this study (i) proposes the use of classical biophysical measurements of water dynamics on germinating diaspores to study germination *sensu stricto*, (ii) points out the best measurement to gauge the germination process, (iii) demonstrates algebraically the polymodal character of the germination process, and (iv) explains how these measurements can be used in practice by scientists.

## 2. Material and Methods

### 2.1. Biological Model and Classification of Diaspore Samples

We used diaspores of a species with great social-economical relevance around the world. We studied caryopses of P44446H transgenic maize hybrid (*Zea mays* L. hybrid type), Caiano cultivar maize (*Zea mays* L. creole type), and BRS254 cultivar wheat (*Triticum aestivum* L.); seeds of NA5909RR cultivar transgenic soybean (*Glycine max* L.); BRS-Horizonte cultivar common bean (*Phaseolus vulgaris* L.); and cypsela of Helio 360 hybrid sunflower (*Helianthus annuus* L.). These diaspores did not possess any obstacles to germination. The initial moisture content of the diaspores was standardized to 11%, independently of physiological quality or species. This standardization was performed based on the lowest moisture content observed in the different samples.

Private companies and research institutions provided the diaspore samples without any chemical treatment. All diaspores, independent of the sample or species, were produced in the 2013/14 crop season in Brazil. The cultivation and harvesting procedures followed those defined by the protocol of each donor company. The diaspores of each species were supplied by the same company. The physiological quality of the samples was evaluated in pretesting, quantifying germinability, viability, and time to the first germination. From this, we studied three samples (with low, medium, and high quality) of each species or cultivar, as is the case of maize. The sample processing and designations low, medium, and high follow the ones proposed by Ribeiro-Oliveira and Ranal [14, 38], who defined them based on viability and germination measurements. Samples composed by diaspores with viability (*V*) and germinability (*G*) higher than 90% were considered high quality, whereas *V* ≤ 60% and *G* ≤ 50% were considered low quality. The diaspores with medium quality had intermediate values for both characters. Thus, we had a mathematical boundary for classifying physiological quality and, therefore, providing a robust study model. This also allowed us to comply with the International Seed Testing Association (ISTA) methods for seed testing validation [39], and it also gave us an important base to discuss the diaspore sample from a biological system point of view. It is important to note that ISTA, based on ISO [40], determines that any method of diaspore testing can only be validated when using samples with different physiological standards.

### 2.2. Water Dynamics on Germinating Diaspores

Diaspores were sowed in germination boxes (plastic boxes) over paper soaked with distilled water (volume in mL equivalent to six times the mass of the paper in g) and then placed on a laboratory bench at 25.3 + 1.5°C under white fluorescent light (11.29 ± 2.84*μ*molm^−2^s^−1^ Photosynthetic Photon Flux Density (PPFD)). The environmental temperature and humidity were regulated by sensors coupled to an air conditioner (Inverter LG–split inverter model, 31 000 BTUs for 41 m^2^). When sowing, we used tweezers to place the diaspore in a specific position, which was maintained until the last assessment. By using the germination paper as a spatial reference, diaspores from monocots were put with the scutellum facing up, whereas those from eudicots were put with the hilum at an angle of 90°. These positions guaranteed that 50% of the diaspore surface was maintained in water, independently of intra- and (or) interspecific morphologies (flatter or rounder). The initial water volume (lost by either experimental manipulation or possible evaporation) was maintained by adding 1 mL (defined in pretesting) of distilled water in the germination box after each mass recording. The diaspore did not suffer anoxia or hypoxia, since the water volume was not sufficient for submersion. These diaspores (*n* = 50) were individually weighed on a digital scale (at 0.0001 g precision) every hour until two hours after embryo protrusion (i.e., germination*sensustricto* + immediatepostgermination). These two hours were minimum-development-time for diaspore-seedling (immediate postgermination) transition of our biological models, including diaspores with “two-step germination” ([41] sense), such as caryopses of maize and wheat. Before mass recording, we removed any water excess (or water film) from the diaspores with a light touch of a paper towel. The germination boxes were opened just for the mass recording of the diaspore individual, assuring the germination boxes a system similar to a humidity chamber (high vapor pressure). The water dynamics on germinating diaspores was measured by means of this recording of mass over time.

### 2.3. Physiological Measurements

We maintained the diaspores used in the water dynamics assessment for 48 hours in the same experimental conditions to analyze them in relation to germinability (*G*; percentage of germination) and viability (*V*). The viability was calculated using the proportion of viable diaspores (evaluated by means of 2,3,5-triphenyl-2H-tetrazolium chloride solution (TTC)) in relation to the total of diaspores analyzed [42]. These data were transformed into percentages. Germinability and viability data were analyzed according to statistical methods proposed by [43] for germination and emergence studies in small samples. Thus, the Student *t*-test (*α* = 0.05) was used considering the proportion of germinated or viable diaspores. This method is based on the statistics for proportions [44]. In this sense, a data set of binomial character can be analyzed by means of the Student *t*-test when it has approximate to normal distribution, which occurs when $n_{1}{\hat{p}}_{1}>5$, $n_{1}{\hat{q}}_{1}>5$, $n_{2}{\hat{p}}_{2}>5$, and $n_{2}{\hat{q}}_{2}>5$, where ${\hat{p}}_{1}=X_{1}/n_{1}$, ${\hat{q}}_{1}=1-{\hat{p}}_{1}$, ${\hat{p}}_{2}=X_{2}/n_{2}$, and ${\hat{q}}_{2}=1-{\hat{p}}_{2}$; ${\hat{p}}_{1}$ and ${\hat{p}}_{2}$ are the proportions of germinated diaspores in sample 1 and sample 2, respectively; ${\hat{q}}_{1}$ and ${\hat{q}}_{2}$ are the proportions of nongerminated diaspores in sample 1 and sample 2, respectively; *X*_1_ and *X*_2_ are the numbers of germinated diaspores in sample 1 and sample 2, respectively; *n*_1_ and *n*_2_ are the sample sizes (total diaspores sowed) in sample 1 and sample 2, respectively. As our data set demonstrated approximate to normal distribution, we compared the samples using the mathematical expression for the Student *t*-test for proportions.

(1) $$\begin{array}{c} t=\frac{{\hat{p}}_{1}-{\hat{p}}_{2}}{\sqrt{\left({\hat{p}}_{1}{\hat{q}}_{1}/n_{1}\right)+\left({\hat{p}}_{2}{\hat{q}}_{2}/n_{2}\right)}}, \end{array}$$

where ${\hat{p}}_{1}$ and ${\hat{p}}_{2}$ are the proportions of germinated diaspores in sample 1 and sample 2, respectively; ${\hat{q}}_{1}$ and ${\hat{q}}_{2}$ are the proportions of nongerminated diaspores in sample 1 and sample 2, respectively; and *n*_1_ and *n*_2_ are the sample sizes (total diaspores sowed) in sample 1 and sample 2, respectively.

### 2.4. Modeling Water Dynamics on Germinating Diaspores

The central point of these measurements and the base of the quantitative treatment is *m*, the water mass over time. The tabulated values of the normalized mass *m*(*t*) were obtained from total mass *M*(*t*) divided by initial (dry) diaspore mass (*M*_0_). First, we submitted the data set to the bootstrap method with 1 000 resamplings, since values generated above this number are similar according to the convergence test. This was an assumption to calculate weighted mass curves and then means and curves of velocity and acceleration. The bootstrap is a technique of successive resampling from original data [45], which ensures that analytical models are reproducible and reliable. The technique is useful in applications for which analytical confidence intervals are unobtainable or when robust nonparametric confidence intervals are required [46]. In addition, the bootstrap easily estimates the normal distribution of an estimator, reduces impacts of the outlier and numerical anomalies, and calculates the estimates of standard error and population parameters of confidence intervals [47]. To calculate these confidence intervals, we used the Algorithm AS 214 for Fortran [48], which is recommended to perform the Monte Carlo confidence intervals.

The first step in the numerical calculation is the interpolation of *m*(*t*) by cubic splines [48]. The tabulated mass measurements in time are interpolated by the expression

(2) $$\begin{array}{c} m=Am_{j}+Bm_{j+1}+Cm_{j}^{\prime \prime }+Dm_{J+1}^{\prime \prime }, \end{array}$$

where the coefficients are

(3) $$\begin{array}{c} A=\frac{t_{j+1}-t}{t_{j+1}-t_{j}},\\ B=\frac{t-t_{j}}{t_{j+1}-t_{j}},\\ C=\frac{1}{6}\left(A^{3}-A\right){\left(t_{j+1}-t_{j}\right)}^{2},\\ D=\frac{1}{6}\left(B^{3}-B\right){\left(t_{j+1}-t_{j}\right)}^{2}. \end{array}$$

Here, *m*^″^is the second derivative of *m*. The cubic spline interpolation keeps the function and its second derivative continuous. This provides a smooth function of the mass increment as a function of time and permits a numerical differentiation of the function to calculate the velocity, defined as the variation of mass over time, which can be written as

(4) $$\begin{array}{c} v=\frac{dm}{dt}=m^{\prime }, \end{array}$$

and acceleration, defined as the variation of velocity over time, which can be written as

(5) $$\begin{array}{c} a=\frac{d^{2}m}{{dt}^{2}}=m^{\prime \prime }. \end{array}$$

The interpolation scheme also provides a simple way to integrate the data and calculate the average values *v*_*m*_ and *a*_*m*_ for velocity and acceleration, respectively. The time average *a*_*m*_ of a parameter *a*(*t*) is defined by the expression

(6) $$\begin{array}{c} a_{m}=\frac{1}{\tau }\int_{0}^{\tau }a\left(t\right)dt, \end{array}$$

where *τ* is the last time tabulated for each species.

The weighted mass of the water dynamics on germinating diaspores was obtained from mass data collected over time and weighted by initial mass (dry mass). We proposed the initial diffusion coefficient, velocity, and acceleration to study water dynamics on germinating diaspores. Raw data of mass were used to calculate the coefficient of initial diffusion, which is a function of the water diffusion ratio by the diaspore radius (*D*/*ρ*^2^; *n* = 50). This ratio was used focusing on a practical sense, since we used cultivated diaspores that, in general, have similar dimensions (see details in supplementary material, Table 1S) for each species. We parameterized *D*/*ρ*^2^ since errors from approximations from diaspore shapes are partially compensated (see more in [49]). We used the diffusion coefficient per unit area as an initial diffusion coefficient because it makes the parameter comparable for species with different diaspore sizes.

**Figure 1:**
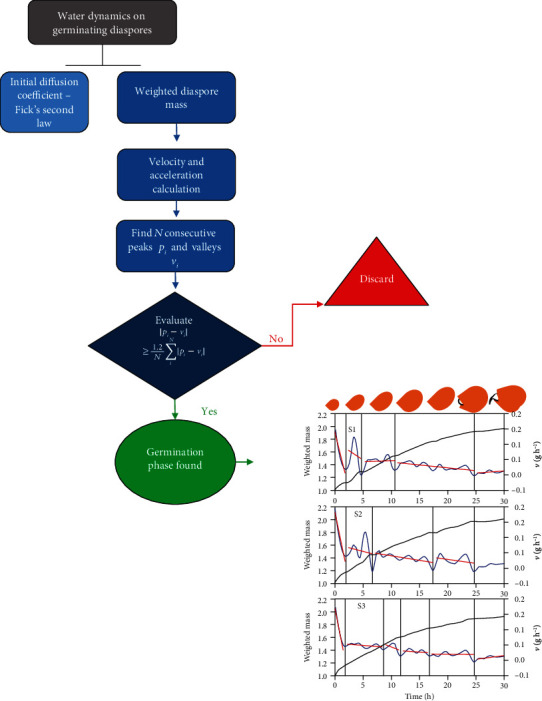
Flowchart of the algorithm used to define different diaspore-seedling phases.

The diffusion ratio coefficient, the square of the diaspore radius *D*/*ρ*^2^, and the saturation mass *M*_∞_ were fitted for a series solution of the Fick differential equation for a spherical model of the diaspore through the expression [50].

(7) $$\begin{array}{c} \frac{M\left(t\right)}{M_{\infty }}=1-\frac{6}{\pi^{2}}\overset{\infty }{\underset{n=1}{\sum }}\frac{1}{n^{2}}\exp\left(-Dn^{2}\pi^{2}\frac{t}{\rho^{2}}\right), \end{array}$$

where *M*(*t*) is the total mass of the diaspore at a time *t*, *D* is the diffusion coefficient, *t* is the time, *n* is a constant (we assumed the constant is equal to 1), and *a* is the radius of the diaspore. The values of *D*/*ρ*^2^ and *M*_∞_ were obtained by a nonlinear fitting, using the Levenberg-Marquardt method [49]. The points used for fitting these parameters correspond to those of the first hours of the experiment, limiting them to the imbibition period called “Phase I” in the results. In equation (7), the total mass is used instead of the normalized one. This is just to account correctly for the boundary conditions imposed to use equation (7) as a solution of the Fick differential equation. We highlight that, firstly, the statistical comparison of biophysical results was based on the bootstrap and the Monte Carlo confidence intervals (see more details at the beginning of this subsection). Secondly, here, pronounced inflections of the velocity curves were used to demonstrate a new germination phase, detected by means of an algorithm that plots linear regressions for each phase (Figure 1). Two criteria were used to detect boundaries of the phases: the first was the velocity peak as a boundary of the first germination phase (imbibition *per se*, where Fick's law might be used), and the second was the differences between peaks and valleys greater than 1.2 times the average of the peak valley differences, since they represent abrupt changes in curve oscillations. The linear regression model for each physiological phase possesses as notation $\hat{v}=\beta_{0}+\beta t$, where *β*_0_ and *β* are parameters with statistical significance (*P* < 0.05) by hypothesis testing based on confidence intervals at 0.05 significance (*α* = 0.05) ($\hat{v}$: estimative for velocity for each germination phase; *β*_0_ and *β*: initial velocity and increment or decrement of velocity for each phase in relation to time (*t*), respectively). To indicate the goodness of fit of the regression model, we used the chi-squared test (*χ*^2^) at 0.01 significance (*α* = 0.01). The model works with normalized values of the derivatives in the attempt to find the main inflections of the curves of weighted mass versus time. The normalization is done by the absolute value of the derivatives. This strategy is used to smooth the natural fluctuations occurring in the measured data and avoid considering abrupt changes arising from experimental errors as a new phase. The figure layout was adjusted according to the resources of Gnuplot (http://www.gnuplot.info/). The Fortran script code used for modeling water dynamics in germinating diaspores (biophysical measurements and germination phase boundaries) is available as supplementary material. From that, the researcher can use or modify the code according to their needs. Finally, we used mean measurements (mean velocity and mean acceleration) and the initial diffusion coefficient of water dynamics to group our biological models by means of Minkowski generalizations of Ward's method in hierarchical clustering [51], one of the most robust and modern methods for clustering. From that, clusters were defined by cutting branches off the dendrogram at 10% of the Minkowski distance.

## 3. Results

**Figure 2:**
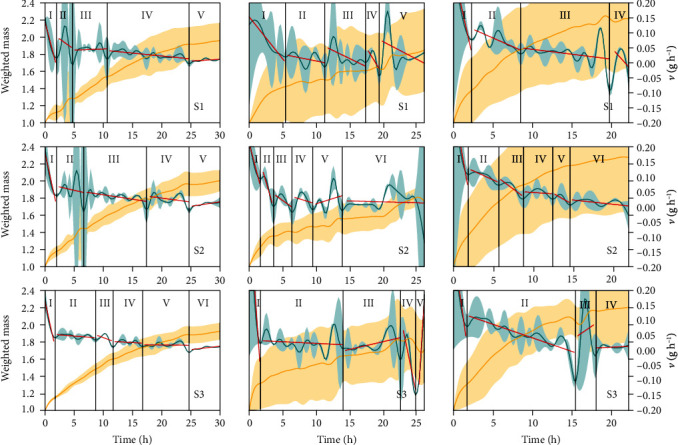
Germination phases (see the vertical black line) determined by the velocity of water dynamics on germinating diaspores of different physiological qualities of the common bean (column 1), sunflower (column 2), and soybean (column 3). S1: sample of low physiological quality; S2: sample of medium physiological quality; S3: sample of high physiological quality. Physiological quality describes viability (*V*) and germinability (*G*) in the sample: high physiological quality is related to a diaspore sample with viability (*V*) and germinability (*G*) higher than 90%, intermediate physiological quality is a diaspore sample with 61% ≤ *V* ≤ 89% and 51% ≤ *G* ≤ 89%, and low physiological quality is a diaspore sample with *V* ≤ 60% and *G* ≤ 50%; curves of weighted mass are represented by yellow lines (see the main axis), while curves of velocity are represented by blue lines (see the secondary axis). Red linear lines represent linear regression ($\hat{v}=\beta_{0}+\beta t$, where *β*_0_ and *β* are parameters with statistical significance (*P* < 0.05) by hypothesis testing based on confidence intervals at 0.05 significance (*α* = 0.05)) ($\hat{v}$: estimative for velocity for each germination phase; *β*_0_ and *β*: initial velocity and increment or decrement of velocity for each phase in relation to time (*t*) that marks each phase). The solid line shows the mean value; the colored area delimits the lower and upper confidence intervals from 1 000 Monte Carlo simulations at 0.05. The embryo protrusion in at least one diaspore occurred two hours before the last recording. *n* = 50 diaspores.

**Figure 3:**
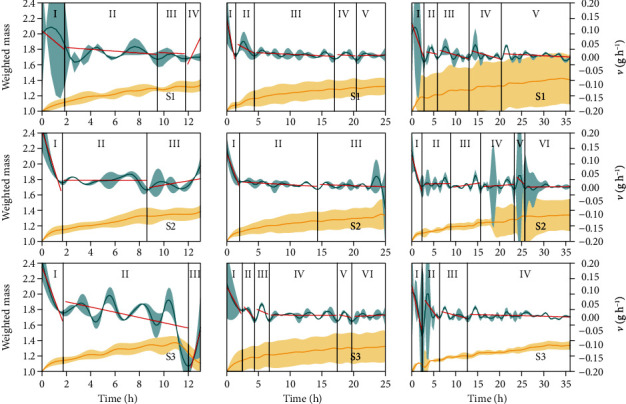
Germination phases (see the vertical black line) determined by the velocity of water dynamics on germinating diaspores of different physiological qualities of wheat (column 1) and maize with low genetic improvement degree (creole type; column 2) and with high genetic improvement degree (hybrid type; column 3). S1: sample of low physiological quality; S2: sample of medium physiological quality; S3: sample of high physiological quality. Physiological quality describes viability (*V*) and germinability (*G*) in the sample: high physiological quality is related to a diaspore sample with viability (*V*) and germinability (*G*) higher than 90%, intermediate physiological quality is a diaspore sample with 61% ≤ *V* ≤ 89% and 51% ≤ *G* ≤ 89%, and low physiological quality is a diaspore sample with *V* ≤ 60% and *G* ≤ 50%; curves of weighted mass are represented by yellow lines (see the main axis), while curves of velocity are represented by blue lines (see the secondary axis). Red linear lines represent linear regression ($\hat{v}=\beta_{0}+\beta t$, where *β*_0_ and *β* are parameters with statistical significance (*P* < 0.05) by hypothesis testing based on confidence intervals at 0.05 significance (*α* = 0.05)) ($\hat{v}$: estimative for velocity for each germination phase; *β*_0_ and *β*: initial velocity and increment or decrement of velocity for each phase in relation to time (*t*) that marks each phase). The solid line shows the mean value; the colored area delimits the lower and upper confidence intervals from 1 000 Monte Carlo simulations at 0.05. The embryo protrusion in at least one diaspore occurred two hours before the last recording. *n* = 50 diaspores.

Wheat caryopses and sunflower cypselas (14% ≤ *G* ≤ 100%; 30% ≤ *V* ≤ 100%) had higher differences in physiological quality, and common bean seeds had lower differences (28% ≤ *G* ≤ 96%; 58% ≤ *V* ≤ 90%) (Table 1). Regardless of intra- and interspecific heterogeneity, the weighted mass pattern remained the same (Figures 2 and 3; supplementary material, Figures 1S–6S), proving the robustness of the measurement to calculate the other biophysical measurements.

In general, the pattern of weighted mass curves seems to be universal; i.e., it is not a species-specific pattern or associated with the physiological quality and degree of genetic improvement (Figures 2 and 3; supplementary material, Figures 1S–6S). On the other hand, velocity and acceleration curves are peculiar to species and may be influenced by the physiological quality and (or) degree of genetic improvement (Figures 2 and 3; supplementary material, Figures 1S–6S). This is emphasized in maize caryopses from the creole type and hybrid type (extremes of the degree of genetic improvement), whose curves only have some differences due to a variety of peculiarities and different physiological qualities (Figure 3; supplementary material, Figures 5S–6S).

As expected, the highest velocity of water influx in diaspores occurs during the first one or two hours from the first contact with water, a period classically associated with imbibition *per se* (this phase (Phase I) is the fastest; Figures 2 and 3; supplementary material, Figures 5S–6S). In general, Phase I is concluded when a sharp decrease in water influx velocity occurs. As water enters the system, the velocity is progressively reduced and, as a consequence, acceleration takes negative values (Figures 2 and 3; supplementary material, Figures 5S–6S). Negative acceleration in this initial phase is explained by the decrease in the driving force of the diffusion effect. However, during the germination process, velocity and acceleration float. In general, velocity fluctuates between 0 and 0.05 mg H_2_O h^−1^ for the common bean seeds, maize (creole and hybrid) caryopses, and wheat caryopses. The fluctuation of the sunflower cypselas has larger values (Figures 2 and 3; supplementary material, Figures 5S–6S).

The initial diffusion coefficient is an interesting physical measurement to quantify the first phase, since the type of reserve material does not seem to be a major factor to determine this coefficient (see *D*/*ρ*^2^ of maize caryopses from the creole type and hybrid type, Table 2). This was confirmed in the clustering analysis of measurements, where the initial diffusion coefficient was related to mean velocity (supplementary material, Table 2S, Figure 7S), a typical metabolism measurement. Among the diaspores studied, maize caryopses from the hybrid type have lower resistance to initial diffusion (2.61 · 10^−2^h^−1^ ≤ *D*/*ρ*^2^ ≤ 8.86 · 10^−2^h^−1^); i.e., they have a quick diffusion process. On xthe other hand, soybean seeds possess the highest resistance to initial diffusion (1.38 · 10^−3^h^−1^ ≤ *D*/*ρ*^2^ ≤ 1.83 · 10^−3^h^−1^) (Table 2). This could be related to slow metabolic intensification.

Mean velocity and mean acceleration of water absorption of germinating diaspores can reflect both aspects of species and physiological quality (supplementary material, Tables 3S–4S, Figure 7S). In clustering analysis, samples with low-quality diaspores grouped species and cultivars according to their genetic improvement degree (supplementary material, Figure 6S). In this case, the common bean and creole-type maize formed a group with a lower improvement degree; sunflower and wheat formed a group with an intermediated improvement degree; and soybean and hybrid-type maize formed two isolated groups with a high improvement degree. Apart from that, samples with high-quality diaspores grouped species and cultivars according to the botanical sense of monocots and eudicots, where maize (both the hybrid type and the creole type) and wheat formed one group, whereas the soybean and common bean, with true seeds, formed another and sunflower a third (supplementary material, Figure 7S). These results confirm the trend of the curves in the algebraic sense. Among the diaspores studied, soybean seeds possess, in general, higher values of mean velocity, and maize caryopses from the creole type or hybrid type have smaller values (Table 2). The common bean seeds have higher mean acceleration of water influx, and sunflower cypselas and wheat caryopses have lower acceleration, demonstrated by negative values (Table 2).

A species-specific pattern for the velocity curve is expressed even when few diaspores in the sample have germinability and viability (see confidence intervals for low-physiological quality samples in Figures 1S–6S). Notwithstanding, it is not possible to propose a general model based on the number of phases of germination *sensu stricto* (Figures 2 and 3). The regression models used to classify different physiological phases have great fitness to the data observed in each phase (see the *χ*^2^ value in Table 3). *β*_0_ ranged from -9.35964 to 2.54529, whereas the *β* range was from -0.32109 to 0.36665. What attracts our attention here is the fact that although these high range values contemplate all biological models, the limits were inputted by the sunflower cypsela pattern. In any case, germination *sensu stricto* has from three to six distinct phases associated with sample physiological quality (Figures 2 and 3). The first phase (Phase I) duration is the most stable trait in the curves, mainly when observing samples with intermediate and high physiological qualities (Figure 2; Table 3). In this case, diaspores of sunflower and wheat have the fastest first germinatiosn phase (1.6 hours), whereas caryopses from hybrid-type maize are slower (2.5 hours). For most species, the last phase is concluded in a sharp peak that occurs with embryo protrusion, demonstrating a completely different metabolic process from this moment on.

## 4. Discussion

Diaspore germination and postgermination are two of the most interesting biological models for studies on plant development patterns, such as those related to plant stress [52]. Therefore, improvements to the tools for experimental analysis of water dynamics on germinating diaspores must be encouraged [1]. Taking this into account, our findings support the idea that the view of germination *sensu stricto* and postgermination as a biological process with three standardized phases is too generic. One similar consideration was made by [30], who split the second phase of germination. However, this approach was only empirical. Thus, we propose a new approach using modeling from classical biophysical measurements to promote robust physiological insights, which can prove to be a paradigm shift that can help plant physiologists concerned with plant growth and development.

When using the initial diffusion coefficient, you can estimate how fast the diffusion process of early diaspore germination of different species is, which can be done in a comparative and quantitative way. Therefore, from laboratory assessments, you can have an idea about the capacity of species and/or varieties in sensing environmental clues to make decisions regarding early plant development, as well as how much resilience might be expected from diaspores of a species or variety in hydric scarcity. In addition, by using velocity and acceleration (mean and curves), you have a metabolism framework in optimum conditions and, from that, an idea of the ability of the diaspore to control its metabolism. We are considering metabolism inferences based on well-established knowledge of pharmacology, which describes metabolic activity in relation to velocity and acceleration calculations. In a practical view, we know that velocity is associated with the transfer of energy and acceleration with the degree of absorption of this energy [53, 54]. In this context, intense metabolism is related to anabolic and catabolic reactions leading to a reduction in the osmotic potential, acting as a driving force for faster water absorption by the diaspore. On the other hand, when these reactions (anabolism and catabolism) are minimized, the water input is reduced, leading to a greater use of water molecules present in the system, which increases the water influx acceleration. Thus, velocity allows us to infer how water is managed by the embryo metabolism, and acceleration gives us an idea of how the water supply is being tapped by it. Given this reasoning, we used the water dynamics on germinating diaspores to propose a numeric classification of the germination phases. A sharp inflection in the velocity curve can be used to infer drastic changes in metabolism. From this classification, we expect to locate points in the curve that are connected to specific metabolic changes and thus help scientists in refining their studies.

Our theory should be confirmed by biochemical and genetic studies, but the fact that minimum biological activity (see samples with low physiological quality) is sufficient to express a species-specific pattern in a diaspore sample gives us propriety to promote it. The diaspores were treated to produce samples with three physiological potentials. For that, we used thermal stress on samples with high physiological quality provided by the companies. The method is according to statistical and mathematical propositions and complies with ISTA method validation for diaspore testing [39]. However, in nature, cell death is a chronic and gradual phenomenon, which goes through several steps up to total cell inability. Thus, our biophysical measurements can probably observe other behaviors in relation to physiological traits in nontreated diaspores.

On the other hand, to discuss the applicability of our theory, we can observe water dynamics measurements on germinating seeds of soybean. The germination in soybean seeds stands out for its slow intensification in contrast to an intense metabolism after the imbibition phase. Probably, the initial diffusion constraint is associated with water movement being restrained by proteins (the main constituent of the seed), which are highly hygroscopic as pointed out by [55]. When dehydrated, protein molecules are coiled to avoid denaturation, and after contact with water, they restructure. However, after restructuring, these molecules do not limit diffusion and the germination metabolism can be expressed intensely, which can be observed by the wide range in velocity and by the high mean velocity.

Similar to soybean seeds, the germination of sunflower cypselas and wheat caryopses presents large variations, which can be observed by a wide fluctuation in curves, which we related to an intense metabolism. However, in relation to soybean seeds, these diaspores possess a low degree of absorption (see acceleration curves and mean acceleration), indicating a better use of water (i.e., small amounts of water are sufficient for germination). Thus, it is indicative that diaspores of sunflower and wheat are probably more resistant to water stress than soybean seeds. By the way, wheat caryopses expose a need for more studies based on time intervals recorded for each species. Even though different physiological phases were detected for these caryopses, they have fewer phases than other species and Phase I was very similar among the different physiological qualities. This occurred because we probably lost information related to critical moments for the physiological processes. When recording diaspore mass, it was necessary to standardize the method and, therefore, these results give us an opportunity for proposing that the analysis time intervals must be defined according to time to the first embryo protrusion, which is a conservative aspect of the species [37]. In this view, a shorter time for the first protrusion is an indication that a shorter recording interval must be used. For agricultural species, such as wheat, this time reduction is possible because only 7 diaspores are sufficient to make inferences about the water dynamics on germinating diaspores [14]. For other species, it will be necessary to define protocols that are adjustable to their peculiarities.

We also demonstrated the existence of more than three germination phases, detected algebraically by changes in velocity and acceleration of water absorption. The changes in metabolism (velocity and acceleration) were defined using elementary differential calculus techniques, which allow us to study the behavior of functions with the help of derivatives [49, 56]. These techniques allow establishing maximum and minimum points, as well as inflection points [49, 56]. Thus, the germination process can be more complex in the biophysical sense than has been previously described (see[31] and their references). In this sense, our main contribution is determining a boundary numerically for each phase regarding germination *sensu stricto*. In addition, we also determine whether each phase is leaning to increments or decrements in metabolism. This was done by means of linear parameters (*β*_0_ and *β*). The parameters *β*_0_ and *β* represent the initial velocity and the mean variation of velocity in relation to the time of each phase, respectively. Thus, these parameters give us clues about how metabolism occurs in each physiological step of germination *sensu stricto*. This is proved by analyzing oleaginous and starchy diaspores. Oleaginous diaspores possess more negative parameters in general (high energy expenditure during catabolism of reserves), whereas starchy diaspores possess parameters near zero (low energy variation during catabolism of reserves). Apart from that, even though the *β* parameter in the first phase is high and negative for starchy diaspores, its fluctuation values are lower than for oleaginous diaspores. In any case, we are opening precedents for studies focusing on the relation between velocity parameters and metabolism.

We highlight two other important considerations. (i) Diaspore scientists well know that two-phase germination (three-phase germination by incorrectly considering postgermination as part of the process) (e.g., [17]) is the most observed pattern, but it is not a universal pattern. An addendum is that here immediate postgermination data (2 hours after embryo protrusion) were spurious but necessary. These data were modeled together with the last records of germination *sensu stricto*, in the last phase, in order to contemplate all types of germination processes (including two-step germination). This strategy proved to be coherent taking into account that a sharp negative value in the velocity curve is congruent with the new cycle of the plant life, which, in general, leads to negative *β* values. For molecular biologists, which have been using two-step germination in their studies, the recommendation is to use *β* as a numerical determination of the beginning of the last germination phase and *β*_0_ as a numerical marker to find the optimum moment for their fine analysis. Independently, we used diaspores that have been mentioned as a case of two-phase germination (e.g., [10, 57, 58]) to study our hypotheses. Our surprise was that when using the measurements here proposed, two-phase germination was not verified. This occurred because classical Phase I and Phase II were split. In our view, Phase I is a strictly physical phase and, therefore, it must be exclusively studied by Fick's law, which might only be related to diffusion *per se*. Our observation is based on the physical common sense that diffusion is a phenomenon negatively associated with water absorption velocity and positively associated with water absorption acceleration. Taking this into account, the second phase (Phase II) is a predominantly biophysical phase, since it describes the velocity related to metabolism intensification (normally called metabolism resumption and related to cell activity intensification and early synthesis *de novo* of organelles, enzymes, and genes; see [17]). After this phase, the metabolism is associated with anabolism and catabolism of the reserves and the water dynamics on germinating diaspores is maintained by inflections in energy input. Therefore, these subsequent phases are predominantly biochemical. (ii) Velocity of water dynamics on germinating diaspores has been associated with reserve tissue and (or) diaspore size [11, 13, 17, 18, 20]. However, we proposed to study the germination process by means of classical biophysical measurements obtained from weighted mass, which proved to have no relation to species, physiological quality, or degree of genetic improvement. Consequently, the biophysical measurements here proposed suffer no (or minimum) interference from reserve tissue and (or) diaspore size. We recommend that the morphoanatomical characters be analyzed in future researches using the classical biophysical measurements of diaspore germination. For that, we are making available our modeling for water dynamics in germinating diaspores. We highlight that, from a physiological point of view, this is very useful because diaspore germination is influenced by genetic and edaphic environmental traits, mainly those related to mother plants during diaspore development [17]. Therefore, our modeling should be used as a previous step for analysis of diaspore properties (e.g., physiology, anatomy and/or morphology, and molecular biology).

It is important to note that our algorithm is not based on image data, but in a near future, it can be useful for this type of analysis. Thus, we are modeling raw data from the observed one (also called “real data”) and, hence, our estimates are related to physiological phenomena *per se*, not from machine learning or other similar methods used to explore expected data (i.e., “synthetic data”) from a computational model. Therefore, we are not using ground truth data to measure algorithm performance from a ground truth correspondence measure (GCM; [59]) for example. We are using robust statistical tools for a sample having an approximated or unknown data distribution, such as defined by classical authors [44, 45, 60]. To perform a previous treatment, we used a weighted measurement of mass and we used bootstrap to reduce numerical anomalies in our data set. We calculated confidence intervals for comparisons, and to certify our regression fitting, we used the chi-squared test, which has lower values, such as pointed out in Table 1. Based on this statistical sense, we performed our inferences. In addition to this data treatment, we used the cubic spline since it is the simplest way to transform a set of records into a numerical function. The use of the spline takes into account two factors: (a) it is extremely simple to treat these types of functions numerically; (b) no *ad hoc* assumption or previous information is required for data interpolation; the splines will only follow the data. These factors are appropriate to our objective of identifying the different phases. Also, this approach avoids Runge's phenomenon in a numerical interpolation [61]. Finally, the splines were treated in the model by a filter that eliminated all the small oscillations, and only the significant changes were kept for the determination of phase transitions. All this was performed automatically, without human interference. The algorithm was capable of correctly identifying a new germination phase by not taking into account small oscillations observed regarding natural fluctuations from the analysis process in the biological system (the standard deviation of the mean values). Thus, there was no misinterpretation of the data, overfitting, or Runge's phenomenon problems present in this model. Any alternative numerical solution, besides the splines, would require some theory describing the water influx into the biological material to infer possible functions as a solution to interpolation. This is not our focus in this manuscript. Our goal is to offer a simple way to study the water dynamics and provide possibilities to improve physiological statements from seed-seedling transition processes. Possible theories come after this work.

Imbibition is the first and shortest germination phase. As imbibition is a physical process [50, 62–64], Fick's second law (diffusion equation) may describe it, and we demonstrated this by fitting the diffusion behavior to the initial time of the germination process. In physics, the coefficient of diffusion, or diffusivity of mass, is a value that represents the ease of solute movement in a solvent [50, 64]. Our results show this coefficient as a character attributed to the physiological quality, species, and variety. In addition, we also showed that the initial diffusion coefficient is not influenced by reserve tissue, which was evident when analyzing creole-type and hybrid-type maize (same type of dispersal unit, same phylogeny, and same reserve material but a different coefficient of initial diffusion). It is important to report that other authors used Fick's law to explore biophysics in germination (from imbibition to embryo protrusion), such as [11–13]. In these cases, a visual analysis of data observed in relation to the model allows us to consider that Fick's second law in fact has a great fitting of data only in the imbibition phase, as pointed out here. In addition, there are several reports in food engineering demonstrating that Fick's second law is applied only to imbibition (e.g., [65–68]), not to predominantly biochemical processes.

The first point presented here is that the initial diffusion coefficient, which is a physical measurement of the diffusion process (imbibition *per se*) of diaspore germination, velocity, and acceleration in water dynamics on germinating diaspores reflect a species pattern. The second point is that the number of germination phases might be determined by the degree of genetic improvement, and the duration of each one might be determined by the physiological quality. The third point that stands out for us is that germination, expressed by means of water dynamics on germinating diaspores, does not have a unique mathematical model for all species. Thus, generalizations must be avoided. The fourth and last consideration is that velocity (transfer of energy) is theoretically the most interesting measurement to study diaspore germination by means of water dynamics, because it offers great potential for inferences about metabolism, which includes its regression coefficients for each germination phase. Acceleration (degree of absorption of energy) also infers metabolism, but in biological science, there are more examples of velocity measuring metabolism than acceleration. Thus, there is greater understanding and use of velocity than acceleration. Further studies on acceleration can clarify if it offers more advantages for understanding the germination process. In the end, we create a new way of determining the germination phases, characterizing not three but more phases, taking the species into consideration.

## Figures and Tables

**Table 1:** Germinability (*G*) and viability (*V*) of the diaspores with different physiological qualities, used in the analyses of water dynamics.

Species	Diaspore sample^1^	*G* (*%*)^∗^	*V* (%)^∗^
*Phaseolus vulgaris* L.	Low physiological quality	28 c	58 c
	Intermediate physiological quality	60 b	64 b
	High physiological quality	96 a	90 a
*Helianthus annuus* L.	Low physiological quality	14 c	30 c
	Intermediate physiological quality	50 b	53 b
	High physiological quality	100 a	100 a
*Zea mays* L. (creole type)	Low physiological quality	40 c	54 c
	Intermediate physiological quality	68 b	70 b
	High physiological quality	100 a	100 a
*Zea mays* L. (hybrid type)	Low physiological quality	40 c	50 c
	Intermediate physiological quality	70 b	82 b
	High physiological quality	100 a	100 a
*Glycine max* L.	Low physiological quality	20 c	48 c
	Intermediate physiological quality	44 b	62 b
	High physiological quality	92 a	92 a
*Triticum aestivum* L.	Low physiological quality	14 c	32 c
	Intermediate physiological quality	42 b	44 b
	High physiological quality	100 a	100 a

Note: ^1^physiological quality describes viability (*V*) and germinability (*G*) in the sample: high physiological quality is related to a diaspore sample with viability (*V*) and germinability (*G*) higher than 90%, intermediate physiological quality is a diaspore sample with 61% ≤ *V* ≤ 89% and 51% ≤ *G* ≤ 89%, and low physiological quality is a diaspore sample with *V* ≤ 60% and *G* ≤ 50%; ^∗^for each species, values followed by the same letter (column) do not differ by the Student *t*-test (*α* = 0.05) for proportions. *n* = 50 diaspores.

**Table 2:** Coefficient of initial diffusion (*D*/*ρ*^2^), mean velocity (*v*_*m*_), and mean acceleration (*a*_*m*_) of water dynamics on germinating diaspores.

Species	Diaspore sample^1^	*D*/*ρ*^2^ (h^−1^)	*v*_*m*_ (g_H_2_O_h^−1^)	*a*_*m*_ (g_H_2_O_h^−2^)
*Phaseolus vulgaris* L.	Low physiological quality	1.94 · 10^−3^	0.0320 (0.0250; 0.0390)	0.0028 (-0.0048; 0.0044)
	Intermediate physiological quality	2.27 · 10^−3^	0.0335 (0.0294; 0.0326)	0.0081 (-0.0028; 0.0094)
	High physiological quality	2.45 · 10^−3^	0.0309 (0.0276; 0.0341)	-0.0005 (-0.0042; -0.0067)
*Helianthus annuus* L.	Low physiological quality	1.95 · 10^−3^	0.0216 (0.0141; 0.0512)	-0. 0400 (-0. 0251; 0.008)
	Intermediate physiological quality	4.11 · 10^−2^	0.0278 (0.0103; 0.0482)	-0.01130 (-0.0373; 0.0009)
	High physiological quality	1.44 · 10^−2^	0.0274 (0.0127; 0.0421)	-0.0140 (-0.0311; 0.0076)
*Zea mays* L. (creole type)	Low physiological quality	2.93 · 10^−3^	0.0130 (0.0089; 0.0170)	0.0052 (-0.0008; 0.0058)
	Intermediate physiological quality	1.91 · 10^−2^	0.0131 (0.0069; 0.0193)	-0.0089 (-0.0159; -0.0016)
	High physiological quality	2.21 · 10^−2^	0.0096 (0.0051; 0.0210)	-0.0049 (-0.0114; 0.0016)
*Zea mays* L. (hybrid type)	Low physiological quality	5.84 · 10^−2^	0.0091 (0.0020; 0.0199)	-0.0024 (-0.0080; 0.0032)
	Intermediate physiological quality	8.86 · 10^−2^	0.0090 (0.0038; 0.0142)	-0.0036 (-0.0068; -0.0005)
	High physiological quality	2.61 · 10^−2^	0.0088 (0.0076; 0.0101)	0.0013 (-0.0077; 0.0030)
*Glycine max* L.	Low physiological quality	1.38 · 10^−3^	0.0550 (0.0279; 0.0822)	-0.0184 (-0.0391; 0.0298)
	Intermediate physiological quality	1.83 · 10^−3^	0.0578 (0.0278; 0.0882)	-0.0182 (-0.0468; 0.0105)
	High physiological quality	1.57 · 10^−3^	0.0548 (0.0355; 0.0074)	-0.0164 (-0.0310; -0.0019)
*Triticum aestivum* L.	Low physiological quality	3.73 · 10^−3^	0.0259 (0.0202; 0.0360)	-0.0190 (-0.0057; 0.0057)
	Intermediate physiological quality	1.77 · 10^−2^	0.0296 (0.0236; 0.0357)	-0.0123 (-0.0183; -0.0063)
	High physiological quality	8.48 · 10^−3^	0.0085 (0.0012; 0.0158)	-0.0193 (-0.0052; -0.0034)

Note: ^1^physiological quality describes viability (*V*) and germinability (*G*) in the sample: high physiological quality is related to a diaspore sample with viability (*V*) and germinability (*G*) higher than 90%, intermediate physiological quality is a diaspore sample with 61% ≤ *V* ≤ 89% and 51% ≤ *G* ≤ 89%, and low physiological quality is a diaspore sample with *V* ≤ 60% and *G* ≤ 50%; *D*/*ρ*^2^ is a parameter of Fick's second law from a data set to perfect fitting and, hence, without variability measurements. The values of *v*_*m*_ and $\bar{a}$ represent the average (lower confidence interval; upper confidence interval) obtained from 1 000 Monte Carlo simulations at 0.05. *n* = 50 diaspores.

**Table 3:** Velocity models in germination phases of diaspore samples with different physiological qualities.

Species	Diaspore sample^1^	Model^2,3^	Diaspore germination phases^4^					
			I	II	III	IV	V	VI
*Phaseolus vulgaris* L.	Low physiological quality	*β*_0_	0.13634	0.1145	0.03871	0.06153	-0.02462	—
		*β*	0.0745	0.01391	0.00077	0.00193	0.00122	—
		Chi-square^4^	0.0008	0.0139	0.0036	0.0029	0.0002	—
	Intermediate physiological quality	*β*_0_	0.17814	0.07729	0.06946	0.08119	-0.07285	—
		*β*	0.08859	0.00449	0.00296	0.00262	0.00297	—
		Chi-square^4^	0.0012	0.0182	0.0033	0.0048	0.0001	—
	High physiological quality	*β*_0_	0.16433	0.05636	0.11524	0.04371	0.0233	-0.05316
		*β*	0.08863	0.0016	0.00723	0.00114	0.00023	0.00225
		Chi-square^4^	0.0006	0.0006	0.0012	0.0007	0.0019	0.0001

*Helianthus annuus* L.	Low physiological quality	*β*_0_	0.15334	0.05068	0.17678	0.68799	0.30398	—
		*β*	-0.02813	-0.00446	-0.01088	-0.03667	-0.01173	—
		Chi-square^4^	0.0036	0.0070	0.0106	0.0033	0.0519	—
	Intermediate physiological quality	*β*_0_	0.21076	0.27709	0.10099	0.07791	-0.0604	0.02861
		*β*	-0.08535	-0.07786	-0.01614	-0.00692	0.00706	-0.00062
		Chi-square^4^	0.0009	0.0005	0.0024	0.0044	0.0199	0.0545
	High physiological quality	*β*_0_	0.46502	0.03492	-0.07394	2.54529	-9.35964	—
		*β*	-0.32109	-0.00112	0.00521	-0.10833	0.36665	—
		Chi-square^4^	0.0123	0.029	0.0195	0.0117	0.0008	—

*Glycine max* (L.) Merr.	Low physiological quality	*β*_0_	0.30418	0.14099	-0.07804	0.63270	—	—
		*β*	-0.11837	-0.01164	-0.00329	-0.02940	—	—
		Chi-square^4^	0.0043	0.0096	0.0555	0.0081	—	—
	Intermediate physiological quality	*β*_0_	0.20924	0.10542	0.15552	0.13122	0.01778	0.21099
		*β*	-0.06673	-0.0067	-0.01108	-0.00725	0.001	-0.01024
		Chi-square^4^	0.0036	0.004	0.0022	0.0028	0.004	0.0002
	High physiological quality	*β*_0_	0.34092	0.13254	-0.23459	-0.01565	—	—
		*β*	-0.18993	-0.00915	0.01814	0.00131	—	—
		Chi-square^4^	0.0040	0.0275	0.0631	0.0007	—	—

*Triticum aestivum* L.	Low physiological quality	*β*_0_	0.09713	0.04222	0.03629	-1.1405	—	—
		*β*	-0.03737	-0.00341	-0.00204	0.09322	—	—
		Chi-square^4^	0.0004	0.0154	0.0111	0.0004	—	—
	Intermediate physiological quality	*β*_0_	0.21021	0.02604	-0.06925	—	—	—
		*β*	-0.14305	-0.00013	0.00772	—	—	—
		Chi-square^4^	0.0036	0.0097	0.0104	—	—	—
	High physiological quality	*β*_0_	0.18523	0.07666	-2.33769	—	—	—
		*β*	-0.11731	-0.00968	0.17603	—	—	—
		Chi-square^4^	0.0035	0.1802	0.0005	—	—	—

*Zea mays* L. (creole type)	Low physiological quality	*β*_0_	0.13831	0.06939	0.02139	0.03967	-0.00485	—
		*β*	-0.11627	-0.01285	-0.00111	-0.00175	0.00048	—
		Chi-square^4^	0.0008	0.0027	0.0018	0.0009	0.0005	—
	Intermediate physiological quality	*β*_0_	0.12466	0.0225	0.0219	—	—	—
		*β*	-0.06137	-0.00137	-0.00082	—	—	—
		Chi-square^4^	0.0011	0.0018	0.0142	—	—	—
	High physiological quality	*β*_0_	0.11252	0.11184	0.07529	0.00825	0.10183	-0.01969
		*β*	-0.04366	-0.02683	-0.00972	-0.00003	-0.00522	0.00113
		Chi-square^4^	0.0005	0.0004	0.0033	0.003	0.0011	0.0006

*Zea mays* L. (hybrid type)	Low physiological quality	*β*_0_	0.12472	0.0307	0.05606	0.08576	0.02919	—
		*β*	-0.04612	-0.00345	-0.00485	-0.00461	-0.00081	—
		Chi-square^4^	0.0044	0.0015	0.0022	0.0026	0.0047	—
	Intermediate physiological quality	*β*_0_	0.11714	0.01001	-0.02467	-0.01629	0.42651	-0.00371
		*β*	-0.05818	0.00047	0.00261	0.00113	-0.01628	0.00016
		Chi-square^4^	0.0012	0.0016	0.003	0.0018	0.0011	0.0006
	High physiological quality	*β*_0_	0.11763	0.12961	0.04905	0.01612	—	—
		*β*	-0.07313	-0.02234	-0.00419	-0.00041	—	—
		Chi-square^4^	0.0022	0.0056	0.0015	0.0046	—	—

Note: ^1^physiological quality describes viability (*V*) and germinability (*G*) in the sample: high physiological quality is related to a diaspore sample with viability (*V*) and germinability (*G*) higher than 90%, intermediate physiological quality is a diaspore sample with 61% ≤ *V* ≤ 89% and 51% ≤ *G* ≤ 89%, and low physiological quality is a diaspore sample with *V* ≤ 60% and *G* ≤ 50%; ^2^$\hat{v}=\beta_{0}+\beta t$, where *β*_0_ and *β* are parameters with statistical significance (*P* < 0.05) by hypothesis testing based on confidence intervals at 0.05 significance (*α* = 0.05) ($\hat{v}$: estimative for velocity for each germination phase; *β*_0_ and *β*: initial velocity and increment or decrement of velocity for each phase in relation to time (*t*)); ^3^chi-squared value (*χ*^2^) was lower than the theoretical value at 0.01 probability (*P* < 0.01) indicating that model regression has a goodness of fit at 0.01 significance (*α* = 0.01) to describe each germination phase; ^4^germination phases were determined from an algorithm, which detects great inflections from velocity and acceleration curves. *n* = 50 diaspores.

## Data Availability

The data is freely available upon request.

## Supplementary Materials

Supplementary Materials Fortran script code. Table 1S: dimensions (mean ± standarderror) of the studied diaspores (*n* = 50). These diaspores were used as a basis for composing the samples with different physiological qualities. Table 2S: proximity matrix for generalizations of Ward's linkage (agglomeration schedule) in hierarchical clustering of water dynamics measurements on germinating diaspores of different species and/or lineages. Table 3S: the Minkowski distance for generalizations of Ward's linkage (agglomeration schedule) in hierarchical clustering of species and/or lineages from water dynamics measurements of germinating diaspores with low physiological quality. Table 4S: the Minkowski distance for generalizations of Ward's linkage (agglomeration schedule) in hierarchical clustering of species and/or lineages from water dynamics measurements of germinating diaspores with high physiological quality. Figure 1S: inferences from the nonsimulated and simulated (the solid line shows the mean value, the colored area delimits the lower and upper confidence intervals from 1 000 Monte Carlo simulations at 0.05) data sets of weighted mass, velocity, and acceleration curves of water dynamics on germinating seeds with different physiological qualities that belong to the common bean. Figure 2S: inferences from the nonsimulated and simulated (the solid line shows the mean value, the colored area delimits the lower and upper confidence intervals from 1 000 Monte Carlo simulations at 0.05) data sets of weighted mass, velocity, and acceleration curves of water dynamics on germinating cypselas with different physiological qualities that belong to sunflower. Figure 3S: inferences from the nonsimulated and simulated (the solid line shows the mean value, the colored area delimits the lower and upper confidence intervals from 1 000 Monte Carlo simulations at 0.05) data sets of weighted mass, velocity, and acceleration curves of water dynamics on germinating seeds with different physiological qualities that belong to soybean. Figure 4S: inferences from the nonsimulated and simulated (the solid line shows the mean value, the colored area delimits the lower and upper confidence intervals from 1 000 Monte Carlo simulations at 0.05) data sets of weighted mass, velocity, and acceleration curves of water dynamics on germinating caryopses with different physiological qualities that belong to wheat. Figure 5S: inferences from the nonsimulated and simulated (the solid line shows the mean value, the colored area delimits the lower and upper confidence intervals from 1 000 Monte Carlo simulations at 0.05) data sets of weighted mass, velocity, and acceleration curves of water dynamics on germinating caryopses with different physiological qualities that belong to maize caryopses of low genetic improvement degree (creole type). Figure 6S: inferences from the nonsimulated and simulated (the solid line shows the mean value, the colored area delimits the lower and upper confidence intervals from 1 000 Monte Carlo simulations at 0.05) data sets of weighted mass, velocity, and acceleration curves of water dynamics on germinating caryopses with different physiological qualities that belong to maize caryopses of high genetic improvement degree (hybrid type). Figure 7S: dendrogram using Ward's linkage from water dynamics measurements on germinating diaspores of or with (A) different species and/or lineages, (B) low physiological quality, and (C) high physiological quality.
