# Supplementary material for: Water Dynamics on Germinating Diaspores: Physiological Perspectives from Biophysical Measurements
Source: Plant Phenomics. 2020 Dec 6;2020:5196176. doi: 10.34133/2020/5196176 (PMC7869936; doi:10.34133/2020/5196176)
Supplement: Supplementary Materials — Fortran script code. Table 1S: dimensions (mean ± standarderror) of the studied diaspores (n = 50). These diaspores were used as a basis for composing the samples with different physiological qualities. Table 2S: proximity matrix for generalizations of Ward's linkage (agglomeration schedule) in hierarchical clustering of water dynamics measurements on germinating diaspores of different species and/or lineages. Table 3S: the Minkowski distance for generalizations of Ward's linkage (agglomeration schedule) in hierarchical clustering of species and/or lineages from water dynamics measurements of germinating diaspores with low physiological quality. Table 4S: the Minkowski distance for generalizations of Ward's linkage (agglomeration schedule) in hierarchical clustering of species and/or lineages from water dynamics measurements of germinating diaspores with high physiological quality. Figure 1S: inferences from the nonsimulated and simulated (the solid line shows the mean value, the colored area delimits the lower and upper confidence intervals from 1 000 Monte Carlo simulations at 0.05) data sets of weighted mass, velocity, and acceleration curves of water dynamics on germinating seeds with different physiological qualities that belong to the common bean. Figure 2S: inferences from the nonsimulated and simulated (the solid line shows the mean value, the colored area delimits the lower and upper confidence intervals from 1 000 Monte Carlo simulations at 0.05) data sets of weighted mass, velocity, and acceleration curves of water dynamics on germinating cypselas with different physiological qualities that belong to sunflower. Figure 3S: inferences from the nonsimulated and simulated (the solid line shows the mean value, the colored area delimits the lower and upper confidence intervals from 1 000 Monte Carlo simulations at 0.05) data sets of weighted mass, velocity, and acceleration curves of water dynamics on germinating seeds with different physiological qualities tha [file 5196176.f1.zip › 5196176.f1.docx]

**Table 1S.** Dimensions (mean ± standard error) of the studied diaspores (*n* = 50). These diaspores were used as a basis for composing the samples with different physiological qualities.

| Species | Length | Width | Thickness |
| --- | --- | --- | --- |
|  | *………………………mm…………………..* | | |
| *Zea mays* L. (creole-type) | 8.4 ± 0.3 | 5.5 ± 0.2 | 4.0 ± 0.5 |
| *Zea mays* L. (hybrid-type) | 8.0 ± 0.2 | 5.0 ± 0.1 | 3.8 ± 0.3 |
| *Phaseolus vulgaris* L. | 10.1 ± 0.5 | 6.6 ± 0.3 | 5.0 ± 0.3 |
| *Glycine max* L. | 6.9 ± 0.3 | 6.4 ± 0.4 | 5.8 ± 0.3 |
| *Helianthus annuus* L. | 12.4 ± 0.6 | 7.5 ± 0.1 | 3.8 ± 0.4 |
| *Triticum aestivum* L. | 5.0 ± 0.3 | 2.8 ± 0.3 | 2.8 ± 0.2 |


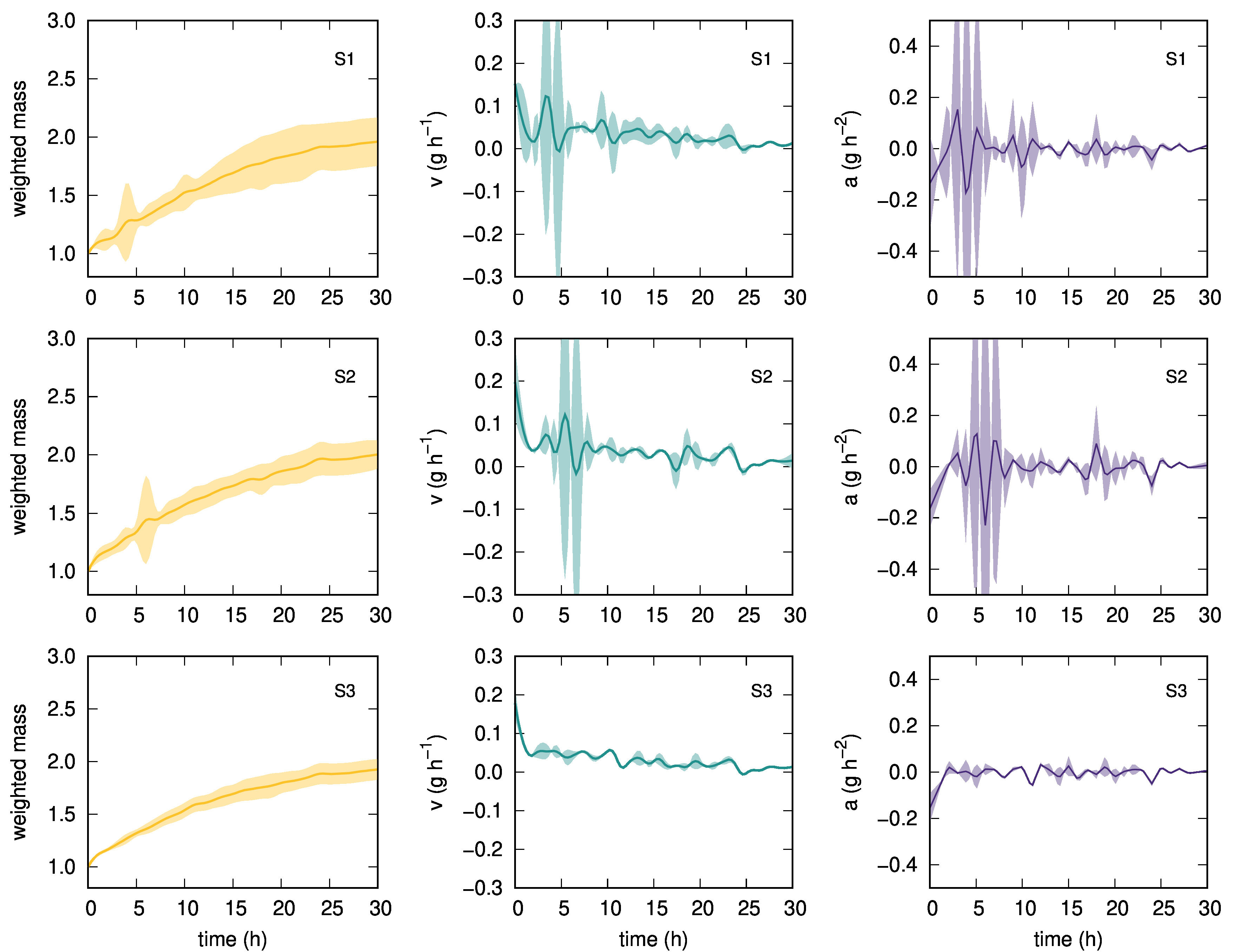


**Figure 1S**. Weighted mass, velocity, and acceleration curves of water dynamics on germinating seeds in different physiological quality belong to common bean. *v*: Velocity of water dynamic on germinating seeds; *a*: acceleration of water dynamic on germinating seeds. S1: Sample of low physiological quality; S2: Sample of medium physiological quality; S3: Sample of high physiological quality. Solid curves represent mean values of inferences from non-simulated data set. The colored area are simulated curves that delimits the lower and upper confidence intervals from 1 000 Monte Carlo simulations at 0.05. The embryo protrusion in at least one seed occurred two hours before the last recording.


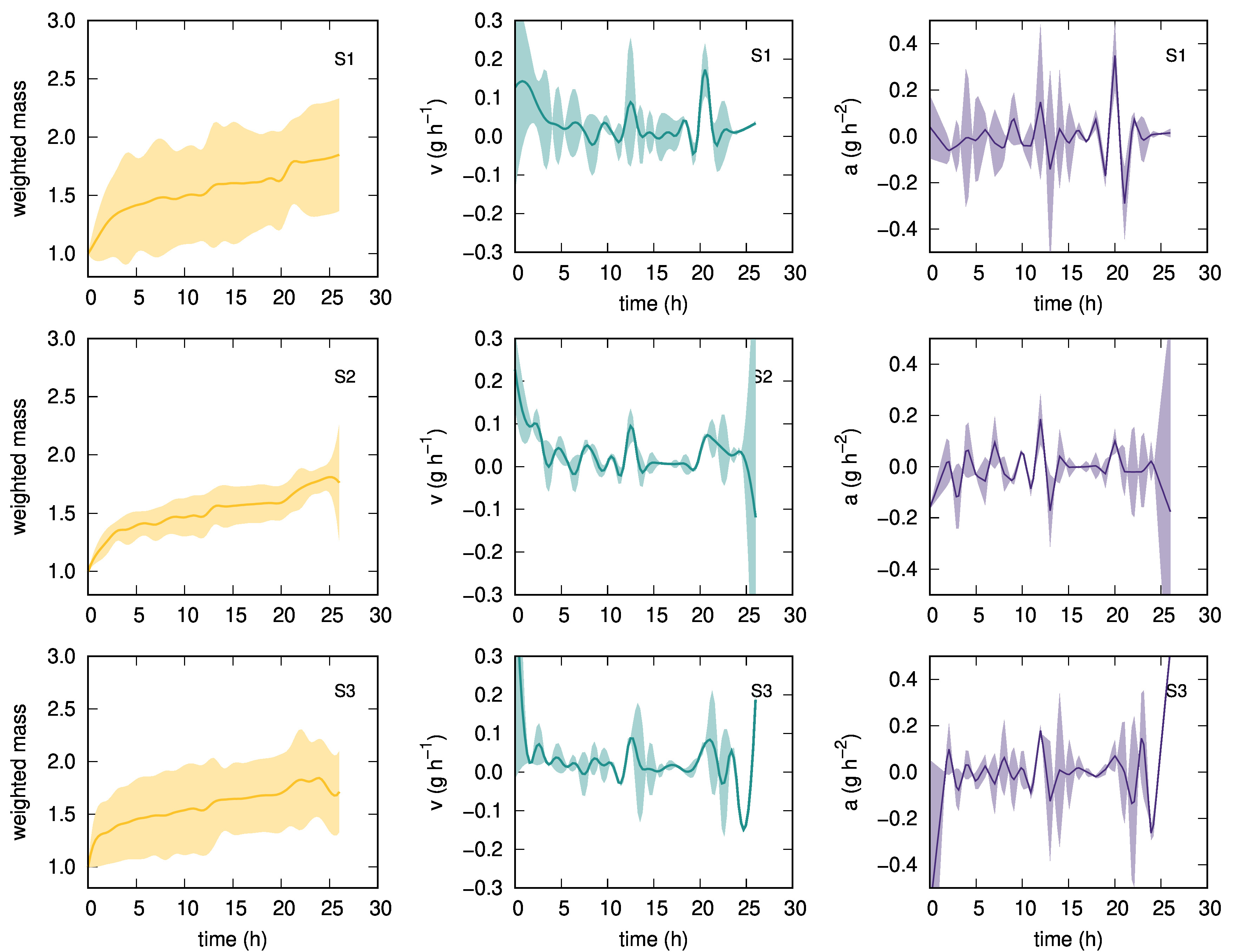


**Figure 2S**. Weighted mass, velocity, and acceleration curves of water dynamics on germinating cypselas in different physiological quality belong to sunflower. *v*: Velocity of water dynamic on germinating cypselas; *a*: acceleration of water dynamic on germinating cypselas. S1: Sample of low physiological quality; S2: Sample of medium physiological quality; S3: Sample of high physiological quality. Solid curves represent mean values of inferences from non-simulated data set. The colored area are simulated curves that delimits the lower and upper confidence intervals from 1 000 Monte Carlo simulations at 0.05. The embryo protrusion in at least one seed occurred two hours before the last recording.


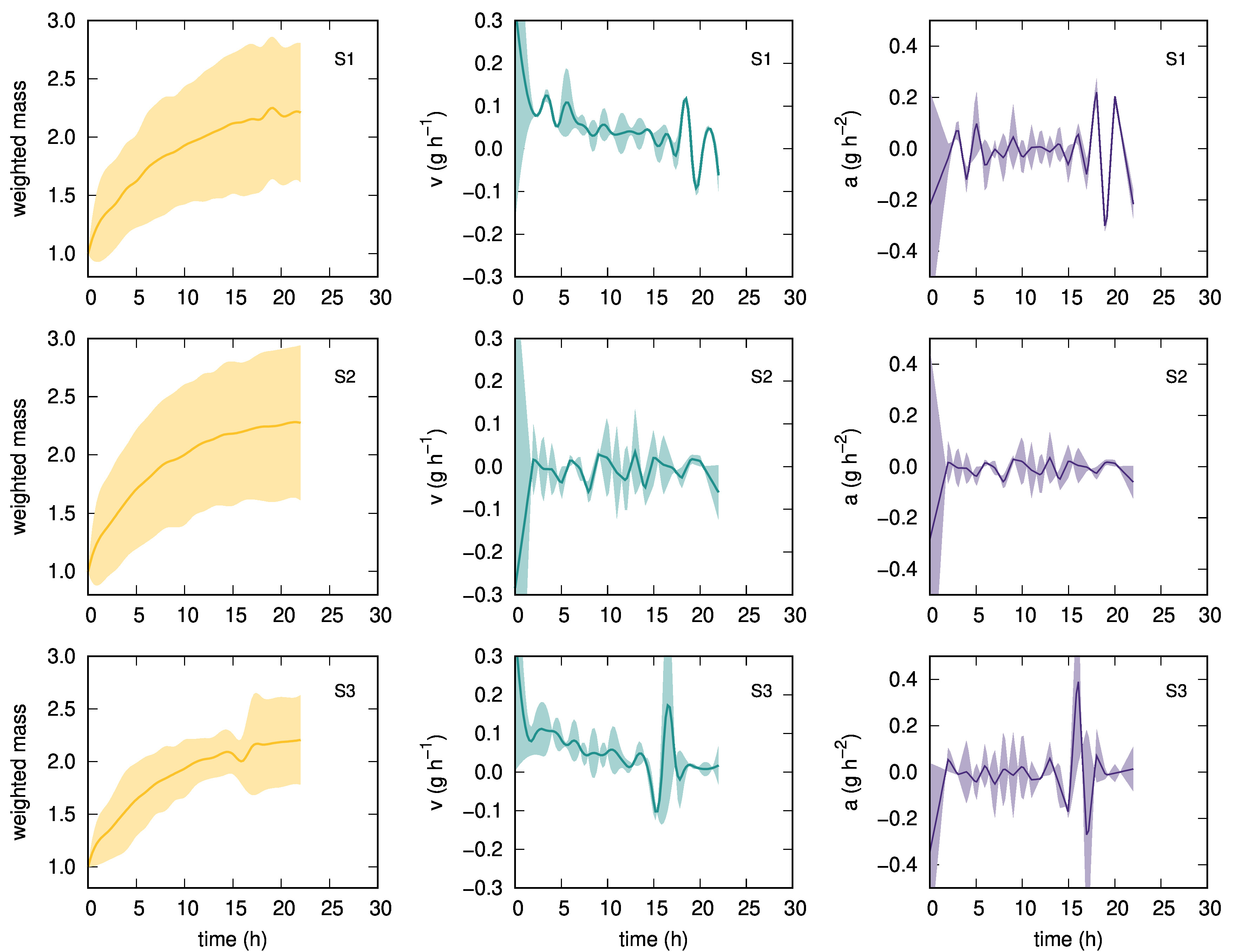


**Figure 3S**. Weighted mass, velocity, and acceleration curves of water dynamics on germinating seeds in different physiological quality belong to soybean. *v*: Velocity of water dynamic on germinating seeds; *a*: acceleration of water dynamic on germinating seeds. S1: Sample of low physiological quality; S2: Sample of medium physiological quality; S3: Sample of high physiological quality. Solid curves represent mean values of inferences from non-simulated data set. The colored area are simulated curves that delimits the lower and upper confidence intervals from 1 000 Monte Carlo simulations at 0.05. The embryo protrusion in at least one seed occurred two hours before the last recording.


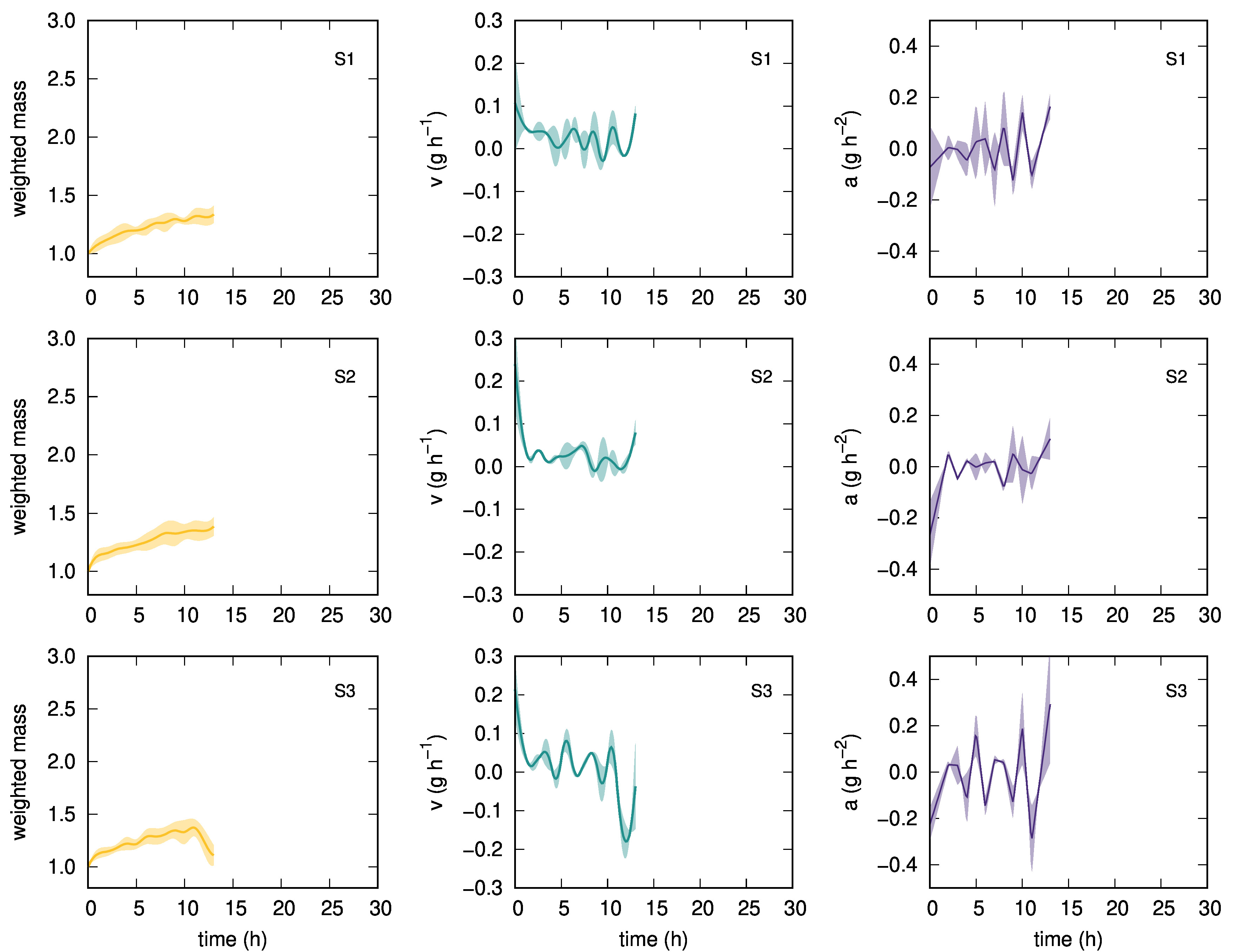


**Figure 4S**. Weighted mass, velocity, and acceleration curves of water dynamics on germinating caryopses in different physiological quality belong to wheat. *v*: Velocity of water dynamic on germinating caryopses; *a*: acceleration of water dynamic on germinating caryopses. S1: Sample of low physiological quality; S2: Sample of medium physiological quality; S3: Sample of high physiological quality. Solid curves represent mean values of inferences from non-simulated data set. The colored area are simulated curves that delimits the lower and upper confidence intervals from 1 000 Monte Carlo simulations at 0.05. The embryo protrusion in at least one seed occurred two hours before the last recording.


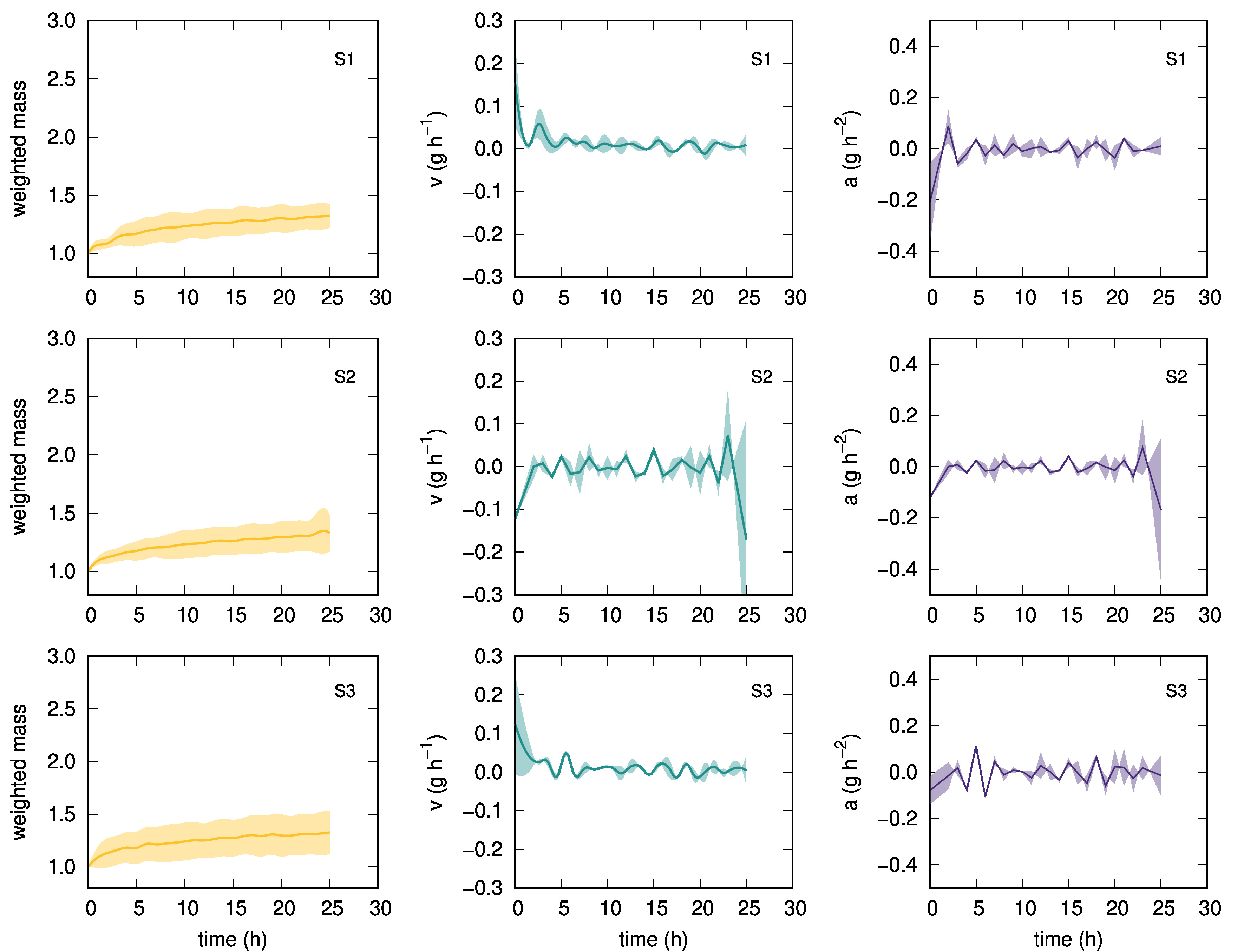


**Figure 5S**. Weighted mass, velocity, and acceleration curves of water dynamics on germinating caryopses in different physiological quality belong to maize caryopses of low genetic improve degree (creole-type). *v*: Velocity of water dynamic on germinating caryopses; *a*: acceleration of water dynamic on germinating caryopses. S1: Sample of low physiological quality; S2: Sample of medium physiological quality; S3: Sample of high physiological quality. Solid curves represent mean values of inferences from non-simulated data set. The colored area are simulated curves that delimits the lower and upper confidence intervals from 1 000 Monte Carlo simulations at 0.05. The embryo protrusion in at least one seed occurred two hours before the last recording.


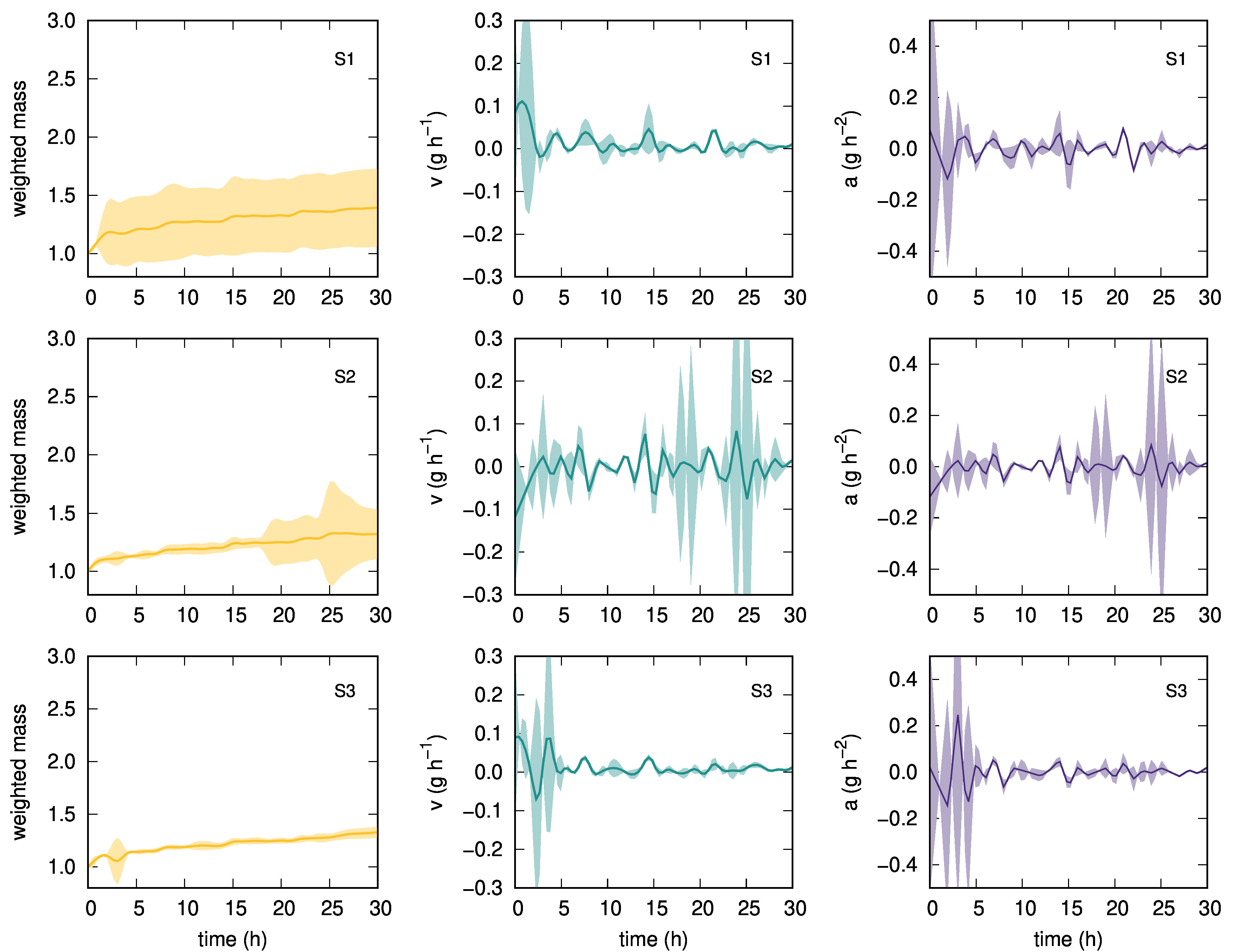


**Figure 6S**. Weighted mass, velocity, and acceleration curves of water dynamics on germinating caryopses in different physiological quality belong to maize caryopses of high genetic improve degree (hybrid-type). *v*: Velocity of water dynamic on germinating caryopses; *a*: acceleration of water dynamic on germinating caryopses. S1: Sample of low physiological quality; S2: Sample of medium physiological quality; S3: Sample of high physiological quality. Solid curves represent mean values of inferences from non-simulated data set. The colored area are simulated curves that delimits the lower and upper confidence intervals from 1 000 Monte Carlo simulations at 0.05. The embryo protrusion in at least one seed occurred two hours before the last recording.

**Table 2S**. Proximity matrix for generalizations of Ward linkage (agglomeration schedule) in hierarchical clustering of water dynamics measurements on germinating diaspores of different species and/or lineages.

| **Case** | | | **Proximity matrix** | | | | | | | |
| --- | --- | --- | --- | --- | --- | --- | --- | --- | --- | --- |
|  |  |  | 1: Initial diffusion coefficient | | | 2: Mean velocity | | 3: Mean acceleration | | |
| 1: Initial diffusion coefficient | | | | 0.000 | | 0.540 | | 0.830 | | |
| 2: Mean velocity | | | | 0.540 | | 0.000 | | 0.890 | | |
| 3: Mean acceleration | | | | 0.830 | | 0.890 | | 0.000 | | |
| **Agglomeration Schedule** | | | | | | | | | | |
| **Stage** | Cluster Combined | | | | Coefficients | | Stage Cluster First Appears | | | Next Stage |
|  | Cluster 1 | Cluster 2 | | |  | | Cluster 1 | | Cluster 2 |  |
| 1 | 1 | 2 | | | 0.027 | | 0 | | 0 | 2 |
| 2 | 1 | 3 | | | 0.076 | | 1 | | 0 | 0 |

**Table 3S**. Minkowski distance for generalizations of Ward linkage (agglomeration schedule) in hierarchical clustering of species and/or lineages from water dynamics measurements of germinating diaspores with low physiological quality.

| **Case** | | | **Minkowski Distance*** | | | | | | | | | | |
| --- | --- | --- | --- | --- | --- | --- | --- | --- | --- | --- | --- | --- | --- |
|  |  |  | 1:*Phaseolus* *vulgaris* | | 2:*Helianthus* *annuus* | | 3:*Zea mays* (creole-type) | 4:*Zea mays* (hybrid-type) | | 5:*Glycine max* | | 6:*Triticum aestivum* | |
| 1:*Phaseolus vulgaris* | | | 0.000 | | 0.043 | | 0.019 | 0.056 | | 0.023 | | 0.022 | |
| 2:*Helianthus* *annuus* | | | 0.043 | | 0.000 | | 0.045 | 0.056 | | 0.033 | | 0.021 | |
| 3:*Zea mays* (creole-type) | | | 0.019 | | 0.045 | | 0.000 | 0.055 | | 0.042 | | 0.024 | |
| 4:*Zea mays* (hybrid-type) | | | 0.056 | | 0.056 | | 0.055 | 0.000 | | 0.057 | | 0.055 | |
| 5:*Glycine max* | | | 0.023 | | 0.033 | | 0.042 | 0.057 | | 0.000 | | 0.029 | |
| 6:*Triticum* *aestivum* | | | 0.022 | | 0.021 | | 0.024 | 0.055 | | 0.029 | | 0.000 | |
| **Agglomeration Schedule** | | | | | | | | | | | | |  |
| **Stage** | Cluster Combined | | | Coefficients | | Stage Cluster First Appears | | | | | Next Stage | |  |
|  | Cluster 1 | Cluster 2 | |  |  | Cluster 1 | | | Cluster 2 | |  |  |  |
| 1 | 1 | 3 | | 0.010 | | 0 | | | 0 | | 4 | |  |
| 2 | 2 | 6 | | 0.020 | | 0 | | | 0 | | 3 | |  |
| 3 | 2 | 5 | | 0.037 | | 2 | | | 0 | | 4 | |  |
| 4 | 1 | 2 | | 0.060 | | 1 | | | 3 | | 5 | |  |
| 5 | 1 | 4 | | 0.097 | | 4 | | | 0 | | 0 | |  |

*This is a dissimilarity matrix

**Table** **4S.** Minkowski distance for generalizations of Ward linkage (agglomeration schedule) in hierarchical clustering of species and/or lineages from water dynamics measurements of germinating diaspores with high physiological quality.

| **Case** | | | **Minkowski Distance*** | | | | | | | | | |  |
| --- | --- | --- | --- | --- | --- | --- | --- | --- | --- | --- | --- | --- | --- |
|  |  |  | 1:*Phaseolus* *vulgaris* | | 2:*Helianthus* *annuus* | | 3:*Zea mays* (creole-type) | | 4:*Zea mays* (hybrid-type) | 5:*Glycine max* | | 6:*Triticum aestivum* |  |
| 1:*Phaseolus vulgaris* | | | 0.000 | | 0.140 | | 0.021 | | 0.024 | 0.024 | | 0.022 |  |
| 2:*Helianthus* *annuus* | | | 0.140 | | 0.000 | | 0.135 | | 0.141 | 0.124 | | 0.121 |  |
| 3:*Zea mays* (creole-type) | | | 0.021 | | 0.135 | | 0.000 | | 0.006 | 0.045 | | 0.014 |  |
| 4:*Zea mays* (hybrid-type) | | | 0.024 | | 0.141 | | 0.006 | | 0.000 | 0.046 | | 0.021 |  |
| 5:*Glycine max* | | | 0.024 | | 0.124 | | 0.045 | | 0.046 | 0.000 | | 0.046 |  |
| 6:*Triticum* *aestivum* | | | 0.022 | | 0.121 | | 0.014 | | 0.021 | 0.046 | | 0.000 |  |
| **Agglomeration Schedule** | | | | | | | | | | | | | |
| **Stage** | Cluster Combined | | | Coefficients | | Stage Cluster First Appears | | | | | Next Stage | | |
|  | Cluster 1 | Cluster 2 | |  |  | Cluster 1 | | Cluster 2 | | |  |  |  |
| 1 | 3 | 4 | | 0.003 | | 0 | | 0 | | | 2 | | |
| 2 | 3 | 6 | | 0.014 | | 1 | | 0 | | | 4 | | |
| 3 | 1 | 5 | | 0.026 | | 0 | | 0 | | | 4 | | |
| 4 | 1 | 3 | | 0.054 | | 3 | | 2 | | | 5 | | |
| 5 | 1 | 2 | | 0.155 | | 4 | | 0 | | | 0 | | |

*This is a dissimilarity matrix


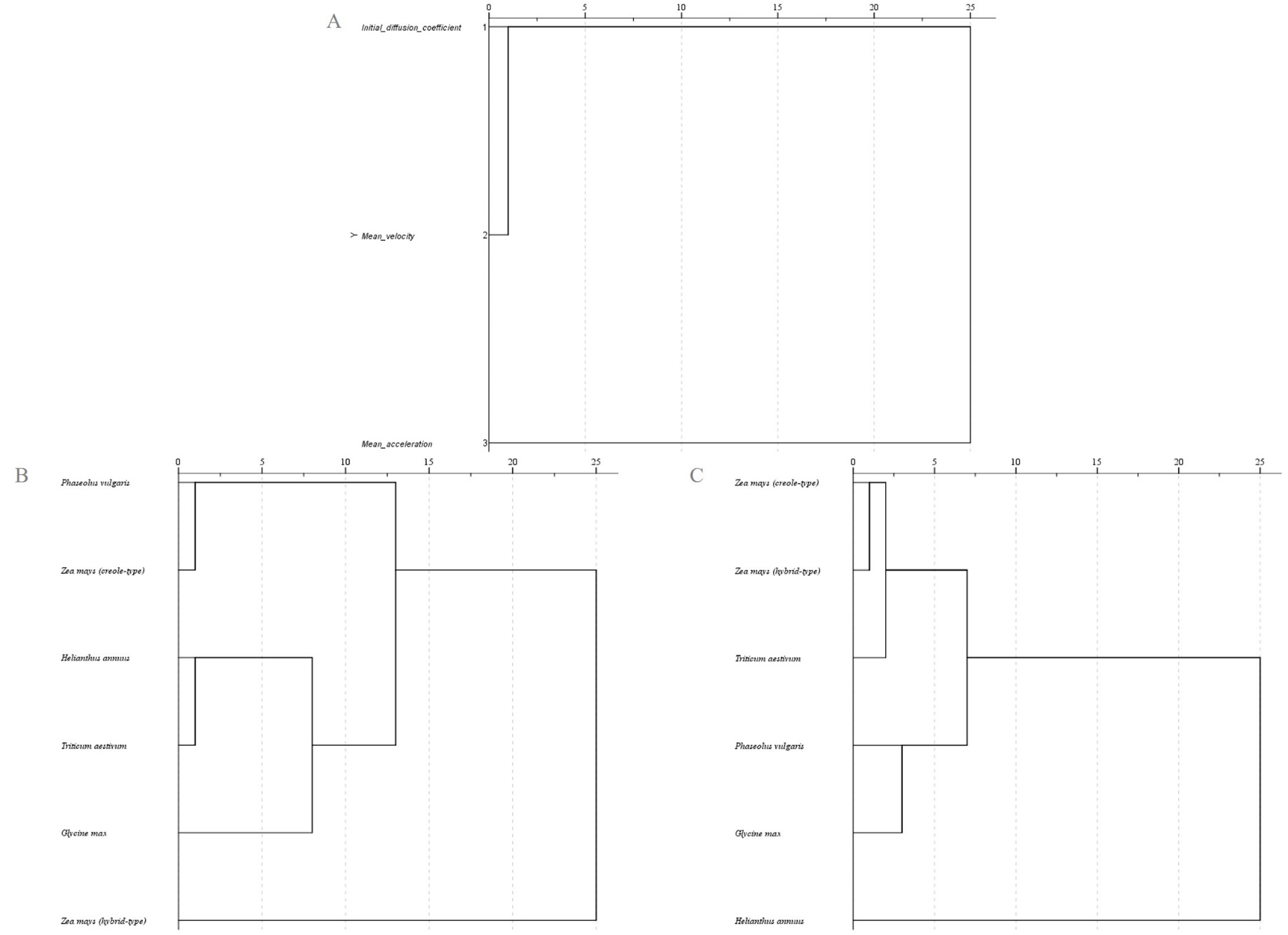


**Figure 7S**. Dendrogram using Ward linkage from water dynamics measurements on germinating diaspores of or with: *A*. different species and/or lineages; *B*. low physiological quality; *C*. high physiological quality.
